# Total health insurance costs in children with a migraine diagnosis compared to a control group

**DOI:** 10.1186/s10194-021-01349-w

**Published:** 2021-11-20

**Authors:** Viola Obermeier, Monika Murawski, Florian Heinen, Mirjam N. Landgraf, Andreas Straube, Rüdiger von Kries, Ruth Ruscheweyh

**Affiliations:** 1 Ludwig Maximilians University Munich, Institute for Social Pediatrics and Adolescent Medicine, Munich, Germany; 2 IFT Institut für Therapieforschung, Munich, Germany; 3 Institute of Health Economics and Health Care Management, Helmholtz Zentrum München - German Research Center for Environmental Health (GmbH), Neuherberg, Germany; 4 Department of Pediatrics, Division of Pediatric Neurology and Developmental Medicine, Ludwig Maximilians University Hospital Munich, Munich, Germany; 5 Department of Neurology, Ludwig Maximilians University Hospital Munich, Munich, Germany

**Keywords:** Health care costs, Migraine, Headache, Children, Claims data

## Abstract

**Background:**

Health care costs of migraine constitute a major issue in health economics. Several publications analyzed health care costs for adult migraine patients, based on questionnaires or secondary (health insurance) data. Although migraine often starts already in primary school age, data on migraine related costs in children is scarce. In this paper we aimed to assess the migraine-related health care costs in 6 to 11 year old children in Germany.

**Methods:**

Using claims data of a large German health insurer (BARMER), overall annual health care costs of 6 to 11 year old children with a diagnosis of migraine in 2017 (*n* = 2597) were compared to a control group of 6 to 11 year old children without a headache diagnosis between 2013 and 2017 (*n* = 306,926). The association of migraine and costs was modeled by generalized linear regression (Gamma regression) with adjustment for sex, age and comorbidities.

**Results:**

Children with migraine caused considerably higher annual per capita health care costs than children without a headache diagnosis (migraine group: € 1018, control group: € 618). Excess costs directly related to migraine amounted to € 115. The remaining excess costs were related to comorbidities, which were more frequent in the migraine group. Mental and behavioural disorders constituted the most expensive comorbidity, accounting for € 105 of the € 400 annual excess costs in the migraine group.

**Conclusion:**

6 to 11 year old children with a migraine diagnosis cause significant direct and comorbidity related excess costs in the German health care system.

**Supplementary Information:**

The online version contains supplementary material available at 10.1186/s10194-021-01349-w.

## Introduction

Health care costs of migraine constitute a major issue in health economics. Several publications analyzed health care costs for adult migraine patients, based on questionnaires [1–3] or secondary (health insurance) data [4–6]. The published mean annual direct health care costs of migraine in adults range from € 86 ^3^ to € 696 (episodic migraine) and € 1495 (chronic migraine) [2] . Annual migraine-related drug costs of € 160 have been reported for German adults [6].

Although migraine often starts already in primary school age [7], data on migraine related costs in children is scarce. An Italian study from 2005 analyzed the direct costs of headache by means of a cost diary in 25 Italian children and adolescents [8]. The average headache-related expense per patient was € 692 over 6 months. An analysis on privately insured children and adolescents in the US in 1999–2000 showed an additional per capita cost of US$ 2872 per year based on 473 children with migraine [9]. A large health care data analysis from the US on children aged 2–17 years with headache found annual per capita excess costs of US$ 687 based on 779 children with headache [10].

Estimation of migraine related health care costs for children using secondary (health care) data faces some specific challenges. First of all the drugs used for migraine treatment in children are not exclusively prescribed for migraine: e.g., ibuprofen and acetaminophen are recommended for the acute treatment of migraine attacks, but also for treatment of tension-type headache [11] and for all other types of pain and the symptom fever. Migraine-specific drugs as triptans are off label in children below 12 years and are used only if ibuprofen and acetaminophen are not efficacious [11]. Therefore, to estimate migraine-related prescription drug costs, the comparison with a non-migraine control group is essential. The same is valid for preventive approaches such as psychology/psychoeducation/psychotherapy and various forms of physiotherapy, which are recommended for the treatment of migraine in children in the German guidelines [11], but are also used in a variety of other disorders.

In Germany, the costs for medical services are settled with the statutory health insurances by lump sums that do not differentiate costs related to specific diagnoses. Thus, identification of migraine related treatment costs can only be achieved by advanced analysis of health insurance data.

Here, we compare a 6 to 11 years old migraine group to a control group with respect to total health care costs in the calendar year 2017. Our analysis of secondary health care data with 309,523 children in total allows an elegant and (due to our large dataset) reliable estimation of both direct and comorbidity related health care costs for migraine in Germany.

## Material and methods

### Data

We used claims data of the BARMER, a statutory health insurer, which covers about 11% of the German population. In Germany, the statutory health insurance benefit catalogue, which also includes the physicians’ settlement per treated patient, the remuneration for diagnostic tests, and most of the prescription drug costs, is specified by the Federal Joint Committee (https://www.g-ba.de/english/), and is identical for all statutory health insurance companies. Supplemental benefits for special patient groups are uncommon. Therefore, individuals base their choice of health insurer more on cost, service and image than on benefits. Indeed, BARMER clients are similar to all persons with statutory health insurance in Germany regarding education level, apart from a slightly higher prevalences of chronic diseases, e.g. diabetes and coronary heart diseases [12]. The BARMER data warehouse provides claims for outpatient physician visits, outpatient hospital care, inpatient treatments, drugs, rehabilitation therapy and medical aids. It includes International Statistical Classification of Diseases, German Modification (ICD-10-GM) codes, billing codes, and Anatomical Therapeutic Chemical (ATC) codes. In addition, patients’ year of birth and sex can be extracted. As the data warehouse provides only pseudonymized data as required by the EU data protection laws, access to source data for case validation was not possible.

### Case definitions

We selected children born in 2006 to 2011 who were continuously enrolled with the BARMER between 2013 and 2017. The migraine group was defined by at least one outpatient physician visit with a diagnosis of ICD-10 G43.x (migraine) in 2017. The German health care system requires further specification of outpatient ICD-10 diagnoses as ‘Z’ = history of diagnosis, ‘A’ = excluded diagnosis, ‘V’ = suspected diagnosis, and ‘G’ = confirmed diagnosis. For the present analysis, only patients with a diagnosis of migraine marked as ‘G’ (confirmed) were selected. The control group of children without headache was defined by absence of any physician contacts with coding of ICD-10 G43 (migraine), G44 (other headache syndromes) or R51 (headache) in the calendar years 2013 to 2017.

For further analyses, we also considered incident cases in the migraine group only. These were defined by at least one confirmed diagnosis of G43 in 2017, but no diagnosis of G43 in the years 2013 to 2016.

### Calculation of costs

We assessed all-cause expenditures in 2017 due to healthcare utilization for each patient within five types of healthcare utilization:

#### Primary care physicians or specialists

Costs for outpatient physician visits, calculated for each type of physician and as a total. Costs for renal dialysis were not included.

#### Outpatient hospital care

Costs due to outpatient hospital visits, also including specialized care in social pediatric centers, university outpatient clinics, and psychiatric departments or institutions.

#### Inpatient care

Costs for inpatient hospital treatment.

#### Rehabilitation therapy and medical aids

Costs for physiotherapy, speech therapy, occupational therapy and medical aids, e.g. walking aids.

#### Drugs

Costs of prescribed drugs as net costs (including discounts) from the health insurance perspective.

#### Headache related drugs

Using ATC codes, we determined three groups of possibly headache/migraine related drugs and calculated costs of painkillers (including triptans), antiemetic drugs and headache preventive drugs.

#### Painkillers (including triptans)

Triptans (Sumatriptan: N02CC01, Naratriptan: N02CC02, Zolmitriptan: N02CC03, Rizatriptan: N02CC04, Almotriptan: N02CC05, Eletriptan: N02CC06, Frovatriptan: N02CC07), Metamizole Sodium: N02BB02, Ergotamine: N02CA02, Tramadol: N02AX01, Tilidine: N02AX02, Ibuprofen (C01EB16, G02CC01, M01AE01, M02AA13, R02AX02) and Paracetamol (N02BE01).

#### Antiemetic drugs

Metoclopramide: A03FA01, Domperidone: A03FA03 and Diphenhydramine: R06AA02.

#### Headache preventive drugs

Amitriptyline: N06AA09, Flunarizine: N07CA03, Topiramate: N03AX11, Metoprolol: C07AB02, Propranolol: C07AA05, Coenzyme Q10: C01EB09, Riboflavin / Vitamin B2: A11HA04, Magnesium: A12CC(01–10,30) und Valproic acid: N03AG01.

To avoid bias from rare but exceptionally costly disorders, e.g. requiring treatment in intensive care units or with excessively expensive drugs, we excluded cases with total costs exceeding the 99% quantile = € 9284 from the analysis. In the control group 3093 children were excluded, in the migraine group 34 children were excluded.

### Confounders

We included sex and age as possible confounders in all analyses. In addition, we performed analyses also adjusting for comorbidities, based on confirmed diagnoses in 2017 and grouped according to the 22 chapters of ICD-10-GM [13].

### Statistical analysis

We calculated the proportion of girls, the mean age and the mean number of different ICD-10 chapters with comorbidities in control and migraine group. Frequencies and relative risks of comorbidities by ICD-10 chapters in control and migraine group were determined. Unadjusted means of per capita costs were determined in the migraine and the control groups.

A generalized linear model (Gamma Regression with log link) was calculated to determine the influence of migraine on the total costs with adjustment for sex and age (Model 1: Basic Model). Gamma regression is a common way to deal with highly skewed response variables, e.g. health care costs [14]. Total costs of zero were set to one euro (gamma regression requires costs > 0). Exponents of the resulting components can be interpreted as factors.

Next, a model with additional adjustment for comorbidities within ICD-10 chapters was calculated (Model2: Comorbidity model). BIC (Bayesian Information Criterion) based variable selection (Forward and Backward, SAS proc. hpgenselect) was used to identify the comorbidity chapters finally included in the model. This model allows to disentangle the estimated excess costs to directly migraine-related costs and costs associated to the higher frequency of comorbidities in the migraine group.

We used recycled predictions to estimate adjusted excess costs of migraine based on both the basic model and the comorbidity model [15, 16]. We estimated 95% confidence intervals for the adjusted migraine related cost differences from 1000 bootstrap replications using the percentile method. We modeled likewise the adjusted disease-related costs for each of the preselected comorbidity chapters and calculated the impact of these comorbidity chapters on cost differences between the migraine and control groups.

We furthermore ran the comorbidity model for primary physician or specialist costs. To assess the impact of a recent migraine diagnosis on costs, we additionally calculated the comorbidity model for total costs for incident migraine cases only. Analysis was performed using SAS Version 9.4 (SAS Institute, Cary, NC, USA).

## Results

In this cross sectional study we compared data of 306,926 children (6–11 years old) without a headache diagnosis (control group) to those of 2597 children with a migraine diagnosis in 2017 (migraine group). In the migraine group, 51.2% had their first migraine diagnosis in 2017 (incident cases).

While the proportion of boys and girls was similar, migraine patients were older than controls (see Table 1).

**Table 1:** Characteristics of the study population. CI: confidence interval

Characteristics	Control group (95% CI)	Migraine group (95% CI)	***P*** value
N	306,926	2597 (51.2% incident in 2017)	–
Girls %	48.7 (48.5,48.9)	47.1 (45.2;49.1)	0.12
Mean age in years	8.4 (8.4;8.5)	9.3 (9.3;9.4)	< 0.0001
Mean number of comorbid ICD-10 chapters	3.9 (3.9;3.9)	5.8 (5.7;5.8)	< 0.0001

In the migraine group, comorbidities from a larger number of different ICD-10 chapters were coded. Compared to controls, conditions from all chapters were more common in migraine patients, e.g. from the chapters “Mental and behavioral disorders” (relative risk RR 1.4), “Diseases of the musculoskeletal system and connective tissue” (RR 2.1) and “Diseases of the nervous system” (headache excluded, RR 2.6) (see Table S1).

### Comparison of raw costs between the migraine and control groups

Table 2 presents the unadjusted costs per capita in the migraine and control groups in 2017, broken down by type of healthcare utilization.

**Table 2:** Comparison of raw costs in 2017 for control group and migraine group by type of healthcare utilization. CI: confidence interval

Type of healthcare utilization	Costs per capita control group € ***N*** = 306,926	Costs per capita migraine group € ***N*** = 2597	Difference € (95% CI)
**Primary care physician or specialist visits**	**270**	**481**	**211 (188;232)**
**Outpatient hospital care**	**52**	**96**	**44 (30;58)**
**Social pediatric centre**	24	41	17 (9;25)
**Psychiatry outpatient clinic**	10	16	6 (1;12)
**Other**	18	38	20 (11;29)
**Inpatient hospital care**	**87**	**190**	**103 (76;31)**
**Rehabilitation therapy and medical aids**	**137**	**141**	**4 (−13;21)**
**Physical therapy**	8	21	13 (8;17)
**Speech therapy**	53	40	-13 (−21;-6)
**Occupational therapy**	43	42	-1 (−9;7)
**Other**	32	38	6 (−4;15)
**Drugs**	**72**	**110**	**38 (29;47)**
**Total healthcare costs**	**618**	**1018**	**400 (352;447)**

Total per capita costs were € 400 higher in the migraine compared to the control group. With higher costs for the migraine group in all categories, the largest difference was due to primary care physician or specialist visits and inpatient hospital care. The difference regarding costs for drugs was smaller. Especially regarding painkillers and other possibly headache-related drugs, costs were low: € 2.7 in the control group (of which € 2.0 for ibuprofen) compared to € 6.7 in the migraine group (€ 4.6 ibuprofen), see Table S2.

Costs for primary care physicians and the different types of specialists are further broken down in Table S3. The highest excess costs in the migraine group were found for pediatricians (+ € 78), general practitioners (+ € 18), specialists treating mental disorders (+ € 32), radiologists (+ € 12), and ophthalmologists (+ € 13). Children in the migraine group also had more frequent physician contacts than the control group, as estimated by case numbers (7.1 case numbers per year in the migraine group, 4.6 in the control group, in the German health care system, each case number stands for one or more physician contacts at the same practice or clinic within 3 months).

### Directly migraine-related vs. comorbidity-related costs

Table 3 provides parameters describing the model for total health care costs on preset exposures (age, sex and migraine) and selected comorbidities. While the basic model with migraine, sex and age yielded a factor of 1.72 (data not shown), the factor decreased to 1.16 after adjustment for comorbidities. The highest factor pertained to mental and behavioural disorders (factor 3.24).

**Table 3:** Gamma regression model factors influencing annual total health care costs: migraine, sex and age were preset, comorbidities selected by the Bayesian Information Criterion (BIC). Exponents can be interpreted as multiplicative cost factors. Estimates with *p* < 0.05 are printed in bold

	Exponents of the coefficients
**Intercept**	**134.16**
**Migraine**	**1.16**
**Sex: Girls**	**0.88**
**Age**	1.00
**Comorbidities**	
Certain infectious and parasitic diseases	**1.14**
Neoplasms (A, B)	**1.10**
Diseases of the blood and blood-forming organs and certain disorders involving the immune mechanism (D50-D90)	**1.16**
Endocrine, nutritional and metabolic diseases (E)	**1.40**
Mental and behavioural disorders (F)	**3.24**
Diseases of the nervous system (G^a^)	**1.88**
Diseases of the eye and adnexa (H00-H59)	**1.32**
Diseases of the ear and mastoid process (H60-H95)	**1.22**
Diseases of the circulatory system (I)	**1.26**
Diseases of the respiratory system (J)	**1.30**
Diseases of the digestive system (K)	**1.24**
Diseases of the skin and subcutaneous tissue (L)	**1.13**
Diseases of the musculoskeletal system and connective tissue (M)	**1.28**
Diseases of the genitourinary system (N)	**1.11**
Congenital malformations, deformations and chromosomal abnormalities (Q)	**1.35**
Symptoms, signs and abnormal clinical and laboratory findings, not elsewhere classified (R^b^)	**1.24**
Injury, poisoning and certain other consequences of external causes (S, T)	**1.39**
Codes for special purposes (U)	**1.64**
Factors influencing health status and contact with health services (Z)	**1.22**

^a^Chapter G Diagnoses without ICD G43 and G44, ^b^ Chapter R diagnoses without ICD R51

Based on the factors resulting from the two models, adjusted mean annual costs for migraine were calculated (Table 4).

**Table 4:** Adjusted mean annual costs in the control group and the migraine group, and excess costs of migraine with Bootstrap 95% confidence interval (CI). Model 1 adjusted for age and sex only, model 2 additionally adjusted for comorbidities

	Adj mean annual costs € (95% CI)	Excess costs € (95% CI)
**Model 1 (Basic)**		
Control group	618 (615;622)	
Migraine group	1060 (1013;1113)	442 (395;494)
**Model 2 (Comorbidity adjusted)**		
Control group	706 (700;711)	
Migraine group	821 (776;865)	115 (72;161)

In the basic model, the excess costs of migraine summed up to € 442. After adjustment for comorbidities the migraine related excess costs were reduced to € 115. Therefore, 29% of the observed cost differences between the two groups can be explained by migraine itself. The remaining excess costs in the migraine group are explained by the more frequent comorbidities in the migraine group. E.g., the excess costs for a comorbidity from the chapter “Mental and behavioural disorders” on average amount to € 807 per affected child (Table S4). In the migraine group, 44% of the children had a diagnosis from this chapter, thus, the average cost of comorbidities from this chapter per child was 0.44* € 807 = € 355, compared to 0.31* € 807 = € 250 in the control group. Thus, € 105 of the total excess costs in the migraine group were due to the higher proportion of children with a comorbidity from the “Mental and behavioural disorders” chapter. Table S4 lists contributions of all comorbidity chapters. Comorbidities from the chapters “Mental and behavioural disorders”, “Codes for special purposes” and “Diseases of the eye and adnexa” explained most of the cost difference between migraine and control group.

We also calculated the comorbidity adjusted model specifically for the outcome *Costs of Primary physician or specialist visits.* Resulting adjusted mean annual costs for migraine were € 70 (95% CI, 47;96), which corresponds to the major part (61%) of the total adjusted excess costs for migraine.

To assess the impact of a recent migraine diagnosis, we calculated the model with adjustment for comorbidity chapters including only the incident migraine cases. This resulted in the same factor for migraine (1.16) and a slightly lower adjusted excess cost for migraine (€ 108 with 95% CI: 50;168).

## Discussion

Main results of the present study are:

- A diagnosis of migraine in 6–11 year old children was associated with an average of € 400 per capita excess health care costs per year, of which € 115 (29%) were directly related to migraine.
- The directly migraine related excess costs were mainly caused by visits to primary care physicians and specialists, while prescribed drugs and radiological or laboratory tests made a much smaller contribution.
- The remaining 71% of excess costs were related to comorbidities, which were more frequent in children with migraine. The most cost intensive (albeit not the most frequent) comorbidities were from the chapter “Mental and behavioural disorders”.

There are only limited published data on costs of headache in children. Data from an Italian survey with 25 children and adolescents (7–18 years old) based on cost diaries indicated that headache is associated with direct health care costs of € 692 over 6 months [8]. Recruitment from a tertiary headache center may explain the higher costs compared to our study which analysed general population data. A large study from the US based on 2016 health care data in children aged 2–17 years revealed an excess cost of US$ 687 per year and child with headache [10], while a US study based on private insurance data from the years 1999–2000 found excess costs of US$ 2872 per year and child with migraine [9]. For adults, in the Eurolight study, assessing subjects between 18 and 65 years mostly drawn from the general population of eight European countries, direct costs of migraine as assessed by a questionnaire were estimated at € 86 per year and person [3].

In our study, the annual per capita excess costs in the migraine group were largely generated by primary care physician and specialist visits (€ 211 in total, corresponding to 53%, of which € 70 were directly migraine-related), with visits to the paediatrician and general practitioner accounting for the largest part. This is consistent with US healthcare data, where 54 to 58% of the excess costs in children with headache were generated by office visits [10] [9].

In contrast, the excess costs for possibly migraine-related prescription drugs were small (€ 4, i.e. 1% of total excess costs and 3% of migraine-related excess costs). One reason for this is that the more expensive drugs for acute treatment (triptans) and all preventive drugs are only very rarely needed in young children and not recommended by the German guideline [11]. Also, the major part of the drugs used for migraine treatment in children (e.g. ibuprofen, acetaminophen and magnesium) are also available over the counter. Therefore, it has to be noted that the present analysis only refers to costs covered by the health insurance, and necessarily underestimates the total drug costs associated with paediatric migraine. In recent US healthcare data, costs from prescription drugs for headache were also not relevant in children (indeed, children with headache had prescription drug costs 2% less than controls) [10], while in a study on privately insured children and adolescents with migraine from the years 1999–2000, there was a significant excess cost for prescription drugs of US$ 1157 per year [9]. In the Italian study, drug costs amounted to ~ 7% of the total direct headache-related costs (~€ 42 in 6 months) [8], which again has to be interpreted in the context of a tertiary headache center population. Adult migraine seems to produce higher medication costs also in the general population, amounting to € 21 per person and year or 24% of the direct migraine costs as estimated in the Eurolight project [3]. Annual medication costs are even higher in selected populations, amounting to € 66–94 in German adults being treated in general or neurological practices [6].

Headache may also produce costs related to special diagnostic tests, especially radiology and laboratory assessments. In the present study, these amounted to € 19 or 5% of the total excess costs in the migraine group. In the Eurolight project, diagnostic tests for migraine in adults amounted to € 19 or 22% of the total direct migraine related costs [3]. The fact that in the present study, incident cases were not more expensive than prevalent cases is consistent with the fact that diagnostic testing, which likely is performed preferentially around the time of diagnosis, is not a major cost factor.

Our analysis showed that a diagnosis of migraine was associated with a higher probability of having a comorbidity diagnosis, most frequently from ICD-10 chapter G (Diseases of the nervous system, headache excluded), D50–90 (Diseases of the blood and blood-forming organs) and M (Diseases of the musculoskeletal system and connective tissue). Previous studies, also those using primary data, have shown that migraine is associated with a high number of comorbidities [17, 18]. Obviously, a higher number of comorbidities will also be associated with a higher cost coming from treatment of these comorbidities. Therefore, we used recycled predictions to differentiate directly migraine-related costs from costs related to the comorbidities. It resulted that only 29% (€ 115) of the total excess costs in the migraine group were related to migraine itself, while the remainder was related to the higher frequency of comorbidities in the migraine group. The most expensive comorbidity according to our analysis, although not the most frequent one, related to chapter F (*Mental and behavioural disorders),* which was 13% points more common in the migraine group. High prevalence and significant excess costs of depression and anxiety have been reported previously, both in children and adolescents [9, 10] and in adults with migraine [19]. There are several possibilities to interpret the present results. As psychotherapy is recommended for treatment of disabling headache in children [11], these children will be seen more often by psychologists and psychiatrists, likely resulting in a larger number of diagnoses from the Mental and behavioural disorders chapter, which might be due to specific psychiatric disorders or (more frequent in the present young age group) developmental disorders. Similarly, frequent visits to the primary physician because of migraine likely facilitates finding and treating other comorbidities. This may result in either appropriate treatment or overtreatment. Therefore, both a higher prevalence of additional disorders in the migraine group and a higher rate of diagnosis and treatment of prevalent comorbidities in the migraine group may contribute to the present results.

### Strength and limitations

A major strength of the present study is the large sample (> 300,000) of children continuously insured from 2013 to 2017, which allowed comparison of children with migraine to a control group free of headache diagnoses between 2013 and 2017 and the adjustment for gender, age and comorbidities.

Approx. 90% of the German population is enrolled in statutory insurance. Even if different German statutory health insurers might differ with regard to the prevalence of chronic diseases, the benefit catalogue and related health care costs is the same for all statutory health insurers. We therefore believe our results to be representative for German children with statutory health insurance, which has an advantage over investigating clinical populations e.g. at tertiary headache centers.

Compared to previous approaches using cost diaries, the present study using claims data likely provides a less biased view on migraine related costs. However, the use of claims data also has disadvantages, because it only captures direct costs reimbursed by the health insurance, but not costs resulting from purchase of over the counter drugs (e.g. ibuprofen, acetaminophen and magnesium, see above) and self-paid therapies. Our data also do not allow to map indirect costs such as generated by childrens’ absenteeism from school and parents’ absenteeism from their workplaces. According to [2] and [3], indirect costs related to loss of productivity represent around 70–93% of the total migraine related costs in adults.

As we calculated migraine related costs in the year 2017 based on migraine diagnoses in 2017, costs might be underestimated because part of the children may have received their first migraine diagnosis only towards the end of 2017.

It is also important to consider that the present study only included costs of children that received a ‘confirmed’ migraine diagnosis. Part of the children who suffer from migraine may not receive a migraine diagnosis because migraine characteristics are less specific in children compared with adults [20]. Furthermore, a substantial part of children with recurrent headache in Germany do not see a physician because of their headaches [21] [22].

Finally, although we included age and sex in all analyses and performed extensive analysis of the impact of comorbidities, there might be other important covariates that could not be considered in the present study. In contrast to the US, the German population is smaller and more homogeneous, and race and geographical origin are usually not included in this type of analysis. It should also be noted that all participants in the present study were insured with the same health insurance company, and therefore had the same access to healthcare.

## Conclusion

Direct health care costs for children were principally caused by visits to primary care physicians and specialists. Only a small part of the costs was due to prescription drugs (but note that over the counter drugs may produce additional costs not captured by the health claims data analysed here). A large proportion of the costs turned out not to be directly related to migraine, but rather to the treatment of comorbidities associated with migraine, which therefore should be a focus of future research and treatment strategies. The fact that the largest proportion of migraine-related costs in children stemmed from visits to paediatricians and general practitioners shows that these are the most promising targets for implementation of migraine prevention strategies in children.

## Supplementary Information

**Additional file 1: Table S1.** Frequencies and relative risk of comorbidities by chapters of ICD-10-GM in the control and the migraine group.

**Additional file 2: Table S2.** Costs of painkillers and other possibly headache-related drugs.

**Additional file 3: Table S3.** Comparison of primary care physician and specialist raw costs in 2017 for control group and migraine group.

**Additional file 4: Table S4.** Excess costs of specific diseases and disease related difference in migraine and control group. * G Diagnosis without ICD G43 and G44,** R diagnosis without ICD R51.

## Data Availability

The datasets generated and analysed during the current study are not publicly available due to data confidentiality of the health insurer Barmer.
