# Supplementary material for: Total health insurance costs in children with a migraine diagnosis compared to a control group
Source: J Headache Pain. 2021 Nov 20;22(1):140. doi: 10.1186/s10194-021-01349-w (PMC8605561; doi:10.1186/s10194-021-01349-w)
Supplement: Supplementary file 1 — Additional file 1: Table S1. Frequencies and relative risk of comorbidities by chapters of ICD-10-GM in the control and the migraine group. [file 10194_2021_1349_MOESM1_ESM.docx]

| **Chapter** | **ICD-10-GM** |  | **Frequency**  **control group** | **Frequency migraine group** | **Relative Risk** |
| --- | --- | --- | --- | --- | --- |
| I | A,B | Certain infectious and parasitic diseases | 0.40 | 0.52 | 1.3 |
| II | C00-D48 | Neoplasms | 0.05 | 0.07 | 1.3 |
| III | D50-D90 | Diseases of the blood and blood-forming organs and certain disorders involving the immune mechanism | 0.02 | 0.04 | 2.1 |
| IV | E | Endocrine, nutritional and metabolic diseases | 0.08 | 0.16 | 2.0 |
| V | F | Mental and behavioural disorders | 0.31 | 0.44 | 1.4 |
| VI | G* | Diseases of the nervous system | 0.03 | 0.07 | 2.6 |
| VII | H00-H59 | Diseases of the eye and adnexa | 0.32 | 0.53 | 1.7 |
| VIII | H60-H95 | Diseases of the ear and mastoid process | 0.18 | 0.21 | 1.2 |
| IX | I | Diseases of the circulatory system | 0.03 | 0.06 | 2.2 |
| X | J | Diseases of the respiratory system | 0.56 | 0.70 | 1.3 |
| XI | K | Diseases of the digestive system | 0.13 | 0.20 | 1.5 |
| XII | L | Diseases of the skin and subcutaneous tissue | 0.22 | 0.30 | 1.3 |
| XIII | M | Diseases of the musculoskeletal system and connective tissue | 0.14 | 0.29 | 2.1 |
| XIV | N | Diseases of the genitourinary system | 0.08 | 0.12 | 1.4 |
| XV | O | Pregnancy, childbirth and the puerperium | 0.00 | 0.00 | - |
| XVI | P | Certain conditions originating in the perinatal period | 0.01 | 0.01 | 1.5 |
| XVII | Q | Congenital malformations, deformations and chromosomal abnormalities | 0.13 | 0.21 | 1.6 |
| XVIII | R** | Symptoms, signs and abnormal clinical and laboratory findings, not elsewhere classified | 0.38 | 0.58 | 1.5 |
| XIX | S,T | Injury, poisoning and certain other consequences of external causes | 0.26 | 0.40 | 1.5 |
| XX | U | Codes for special purposes | 0.16 | 0.32 | 2.0 |
| XXI | V,W,X,Y | External causes of morbidity and mortality | 0.00 | 0.00 | 1.1 |
| XXII | Z | Factors influencing health status and contact with health services | 0.45 | 0.57 | 1.3 |

Table S1: Frequencies and relative risk of comorbidities by chapters of ICD-10-GM in the control and the migraine group

* G Diagnosis without ICD G43 and G44 ,** R diagnosis without ICD R51
