# Supplementary material for: Total health insurance costs in children with a migraine diagnosis compared to a control group
Source: J Headache Pain. 2021 Nov 20;22(1):140. doi: 10.1186/s10194-021-01349-w (PMC8605561; doi:10.1186/s10194-021-01349-w)
Supplement: Supplementary file 2 — Additional file 2: Table S2. Costs of painkillers and other possibly headache-related drugs. [file 10194_2021_1349_MOESM2_ESM.docx]

| **Drug** | **Costs in € per capita control group**  **N=306 926** | **Costs in € per capita migraine group**  **N=2 597** |
| --- | --- | --- |
| **Painkillers**  **Ibuprofen**  **Paracetamol**  **Triptans**  **Other** | **2.3**  2.0  0.2  0.0  0.1 | **6.0**  4.6  0.4  0.5  0.6 |
| **Antiemetic drugs** | **0.0** | **0.2** |
| **Betablockers, Topiramate,**  **Flunarizine,**  **Amitriptyline *** | **0.5** | **0.5** |
| **Total** | **2.7** | **6.7** |

Table S2: Costs of painkillers and other possibly headache-related drugs

* prescribed for migraine prevention or other diagnoses than migraine
