# Supplementary material for: Total health insurance costs in children with a migraine diagnosis compared to a control group
Source: J Headache Pain. 2021 Nov 20;22(1):140. doi: 10.1186/s10194-021-01349-w (PMC8605561; doi:10.1186/s10194-021-01349-w)
Supplement: Supplementary file 3 — Additional file 3: Table S3. Comparison of primary care physician and specialist raw costs in 2017 for control group and migraine group. [file 10194_2021_1349_MOESM3_ESM.docx]

| **Primary care physicians and different types of specialists** | **Costs per capita control group**  **€**  **N=306 926** | **Costs per capita migraine group**  **€**  **N=2 597** | **Difference**  **€** |
| --- | --- | --- | --- |
| Pediatrician | 96 | 174 | 78 (72;84) |
| General practitioner | 30 | 48 | 18 (15;22) |
| Psychiatrist  Psychotherapist  Psychosomatic medicine  Psychological psychotherapist | 41 | 73 | 32 (21;42) |
| Radiology | 2 | 14 | 12 (10;14) |
| Ophthalmology | 17 | 30 | 13 (11;14) |
| Laboratory Medicine | 7 | 14 | 7 (5;9) |
| Surgery | 10 | 18 | 8 (6;9) |
| Neurology | 1 | 3 | 2 (2;3) |
| Others and unknown | 66 | 107 | 41 (25;56) |
| **Total outpatient physician costs** | **270** | **481** | **211 (188;232)** |

Table S3: Comparison of primary care physician and specialist raw costs in 2017 for control group and migraine group
