# Supplementary material for: Total health insurance costs in children with a migraine diagnosis compared to a control group
Source: J Headache Pain. 2021 Nov 20;22(1):140. doi: 10.1186/s10194-021-01349-w (PMC8605561; doi:10.1186/s10194-021-01349-w)
Supplement: Supplementary file 4 — Additional file 4: Table S4. Excess costs of specific diseases and disease related difference in migraine and control group. * G Diagnosis without ICD G43 and G44,** R diagnosis without ICD R51. [file 10194_2021_1349_MOESM4_ESM.docx]

| **Disease or comorbidity chapter** | **Costs of disease per diseased child (€)** | **Proportion of children with disease: control group** | **Proportion of children with disease: migraine group** | **Average costs per child: control group (€)** | **Average costs per child: migraine group (€)** | **Disease related excess costs migraine group compared to the control group (€)** |
| --- | --- | --- | --- | --- | --- | --- |
| **Migraine** | 115 | 0 | 1 | 115*0=0 | 115*1=115 | 115 |
| **Mental and behavioural disorders** | 807 | 0.31 | 0.44 | 807*0.31=250 | 807*0.44=355 | 105 |
| **Codes for special purposes** | 417 | 0.16 | 0.32 | 417*0.16=67 | 417*0.32=133 | 67 |
| **Diseases of the eye and adnexa** | 192 | 0.32 | 0.53 | 192*0.31=61 | 192*0.53=98 | 37 |
| **Injury, poisoning and certain other consequences of external causes** | 252 | 0.26 | 0.40 | 252*0.26=66 | 252*0.40=101 | 35 |
| **Symptoms, signs and abnormal clinical and laboratory findings, not elsewhere classified **** | 154 | 0.38 | 0.58 | 154*0.38=59 | 154*0.58=89 | 30 |
| **Diseases of the musculoskeletal system and connective tissue** | 179 | 0.14 | 0.29 | 179*0.14=25 | 179*0.29=52 | 27 |
| **Diseases of the respiratory system** | 175 | 0.56 | 0.70 | 175*0.56=98 | 175*0.70=123 | 25 |
| **Diseases of the nervous system *** | 614 | 0.03 | 0.07 | 614*0.03=18 | 614*0.07=43 | 24 |
| **Endocrine, nutritional and metabolic diseases** | 267 | 0.08 | 0.16 | 267*0.08=21 | 267*0.16=43 | 22 |
| **Factors influencing health status and contact with health services** | 143 | 0.45 | 0.57 | 143*0.45=64 | 143*0.57=82 | 18 |
| **Congenital malformations, deformations and chromosomal abnormalities** | 227 | 0.13 | 0.21 | 227*0.13=30 | 227*0.21=48 | 18 |
| **Certain infectious and parasitic diseases** | 95 | 0.40 | 0.52 | 95*0.40=38 | 95*0.52=49 | 11 |
| **Diseases of the digestive system** | 131 | 0.13 | 0.20 | 131*0.13=17 | 131*0.20=26 | 9 |
| **Diseases of the skin and subcutaneous tissue** | 93 | 0.22 | 0.30 | 93*0.22=20 | 93*0.30=28 | 7 |
| **Diseases of the ear and mastoid process** | 151 | 0.18 | 0.21 | 151*0.18=27 | 151*0.21=32 | 5 |
| **Diseases of the circulatory system** | 181 | 0.03 | 0.06 | 181*0.03=5 | 181*0.06=11 | 5 |
| **Diseases of the genitourinary system** | 89 | 0.08 | 0.12 | 89*0.08=7 | 89*0.12=11 | 4 |
| **Diseases of the blood and blood-forming organs and certain disorders involving the immune mechanism** | 111 | 0.02 | 0.04 | 111*0.02=2 | 111*0.04=4 | 2 |
| **Neoplasms** | 68 | 0.05 | 0.07 | 68*0.05=4 | 68*0.07=5 | 1 |

Table S4: Excess costs of specific diseases and disease related difference in migraine and control group. * G Diagnosis without ICD G43 and G44 ,** R diagnosis without ICD R51
